# Hepatitis C virus infection is associated with hepatic and adipose tissue insulin resistance that improves after viral cure

**DOI:** 10.1111/cen.13924

**Published:** 2019-01-17

**Authors:** Teegan R. Lim, Jonathan M. Hazlehurst, Andrei I. Oprescu, Matthew J. Armstrong, Sewa F. Abdullah, Nigel P. Davies, Robert Flintham, Peter Balfe, David J. Mutimer, Jane A. McKeating, Jeremy W. Tomlinson

**Affiliations:** ^1^ NIHR Liver Biomedical Research Unit University of Birmingham Birmingham UK; ^2^ CRUK Clinical Trials Unit University of Birmingham Birmingham UK; ^3^ Institute of Metabolism and Systems Research University of Birmingham Birmingham UK; ^4^ School of Sport, Exercise & Rehabilitation Sciences University of Birmingham Birmingham UK; ^5^ Medical Physics University Hospitals Birmingham Birmingham UK; ^6^ Nuffield Department of Medicine University of Oxford Oxford UK; ^7^ Oxford Centre for Diabetes, Endocrinology & Metabolism University of Oxford Oxford UK

**Keywords:** adipose tissue, HCV, hepatic, insulin resistance

## Abstract

**Background:**

Chronic hepatitis C (CHC) is associated with systemic insulin resistance, yet there are limited data on the tissue‐specific contribution in vivo to this adverse metabolic phenotype, and the effect of HCV cure.

**Methods:**

We examined tissue‐specific insulin sensitivity in a cohort study involving 13 patients with CHC compared to 12 BMI‐matched healthy control subjects. All subjects underwent a two‐step clamp incorporating the use of stable isotopes to measure carbohydrate and lipid flux (hepatic and global insulin sensitivity) with concomitant subcutaneous adipose tissue microdialysis and biopsy (subcutaneous adipose tissue insulin sensitivity). Investigations were repeated in seven patients with CHC following antiviral therapy with a documented sustained virological response.

**Results:**

Adipose tissue was more insulin resistant in patients with CHC compared to healthy controls, as evidence by elevated glycerol production rate and impaired insulin‐mediated suppression of both circulating nonesterified fatty acids (NEFA) and adipose interstitial fluid glycerol release during the hyperinsulinaemic euglycaemic clamp. Hepatic and muscle insulin sensitivity were similar between patients with CHC and controls. Following viral eradication, hepatic insulin sensitivity improved as demonstrated by a reduction in endogenous glucose production rate. In addition, circulating NEFA decreased with sustained virological response (SVR) and insulin was more effective at suppressing adipose tissue interstitial glycerol release with a parallel increase in the expression of insulin signalling cascade genes in adipose tissue consistent with enhanced adipose tissue insulin sensitivity.

**Conclusion:**

Chronic hepatitis C patients have profound subcutaneous adipose tissue insulin resistance in comparison with BMI‐matched controls. For the first time, we have demonstrated that viral eradication improves global, hepatic and adipose tissue insulin sensitivity.

## 1 INTRODUCTION

Hepatitis C virus (HCV) infection is a global health problem affecting 170 million people that leads to progressive liver disease including cirrhosis and hepatocellular carcinoma (HCC). Chronic hepatitis C (CHC) is also associated with metabolic syndrome.^1^, ^2^ Type 2 diabetes mellitus (T2DM) is more prevalent in patients with CHC‐associated cirrhosis compared to other causes of cirrhosis.^3^ Metabolic syndrome in CHC is associated with an increased risk of progressive liver diseases^4^ and population‐based studies link T2DM to fibrosis and highlight its role as a risk factor for developing HCC.^5^

Hepatitis C virus replicates within hepatocytes in the liver and yet the sites of insulin resistance in patients with CHC remain elusive, with some studies reporting the liver as the primary site^6^, ^7^ and others suggest extra‐hepatic sites, namely adipose tissue and skeletal muscle.^8^ Most of the current evidence studying insulin resistance in CHC is based on homoeostatic model assessment of insulin resistance (HOMA‐IR) which cannot distinguish sites of IR^9^. Hepatic steatosis and insulin resistance can limit treatment response to interferon‐based therapies.^10^, ^11^, ^12^ Directly acting antiviral agents (DAAs) have recently become the standard of care with high rates of cure >90%.^13^, ^14^, ^15^ Curing HCV infection with DAA or interferon‐based therapies improves the metabolic profile^16^, ^17^, ^18^; however, none of these studies investigated the tissue‐specific effects of HCV infection on IR.

Based upon the premise that CHC is a multisystem disease with a significant metabolic burden, we investigated the tissue‐specific contributions to the adverse metabolic phenotype in CHC using state‐of‐the‐art metabolic phenotyping techniques. Secondly, we assessed the impact of curative treatments on liver function and the adverse metabolic features associated with CHC. Our underpinning hypothesis is that adipose tissue insulin resistance is the cardinal feature of the metabolic abnormalities associated with chronic hepatitis C and this improves with viral eradication.

## 2 PATIENTS AND METHODS

### 2.1 Study subjects

#### 2.1.1 CHC patients

One hundred and two treatment‐naive patients with CHC were diagnosed as anti‐HCV antibody positive and had detectable levels of HCV RNA. Of these, 13 met the inclusion criteria. The majority of the patients were excluded because their treatment was delayed due to the development of directly acting antiviral agents. Eligibility for the study was determined at routine national health service appointment by standard blood tests, clinical history and physical examination/observations to identify coexisting medical illnesses or other contraindications (Supporting Information S1 in Appendix S1). Patients received either interferon‐containing or interferon‐free regimen, which was decided by the clinician following 1‐1 discussion. The types and duration of treatment are listed in Supporting Information S2 in Appendix S1. Of the thirteen patients, 11 received antiviral therapy and two patients did not (contraindications to starting treatment). Of the 11 treated patients, one patient did not achieve sustained virological response (SVR), two patients withdrew consent and one patient was lost to follow‐up, leaving seven patients who completed both parts of the study.

#### 2.1.2 Healthy control subjects

Twelve healthy male volunteers were recruited by local advertisement and provided written informed consent.^19^

### 2.2 Study design and clinical protocol

All subjects were invited to the Welcome Trust Clinical Research Unit where they underwent a two‐step hyperinsulinaemic euglycaemic clamp, adipose tissue microdialysis and biopsy, magnetic resonance spectroscopy (MRS) of the liver and dual energy absorptiometry (DXA) scans to measure body composition and adipose distribution. The first part of the study compared healthy volunteers and CHC patients prior to treatment. Following successful antiviral treatment, seven cured patients were reinvited to undergo a similar metabolic study (second part of study). There was a washout period of at least 3 months after the end of treatment before the metabolic study was performed (Supporting Information S3 in Appendix S1).

#### 2.2.1 Hepatic de novo lipogenesis

The evening prior to the clinical study, subjects visited the research facility and were given a standardized evening meal after which they fasted until the end of the clamp at 14:00 hours the next day. To determine the rates of de novo lipogenesis (DNL), they were given oral ^2^H_2_O (3 g/kg deuterated water in 2 divided doses) at 5 and 10 pm, followed by drinking water enriched to 0.4%.

#### 2.2.2 Two‐step hyperinsulinaemic euglycaemic clamp

At 08:00 hours the following morning, fasting bloods were taken prior to starting the two‐step clamp procedure using established methodology.^19^ A bolus of ^13^C‐glucose (CK Gas Ltd, Hook, UK) was administered (2 mg/kg) over 1 minute followed by constant rate infusion of ^13^C‐glucose (20 µg/kg/min; basal phase). Two hours after starting the clamp, a soluble insulin infusion (Actrapid; Novo Nordisk, Copenhagen, Denmark) was commenced (20 mU/m^2^/min, low insulin to partially suppress hepatic glucose output), together with an infusion of 20% glucose enriched with ^13^C‐glucose to 4%, beginning at 2 mg/kg/min through the same line. Arterialized blood samples were taken at 5‐minute intervals, and the glucose infusion rate changed to maintain fasting glycaemic levels. After a further 2 hours, the insulin infusion rate was then increased to 100 mU/m^2^/kg (high insulin to maximally drive glucose uptake) for the final 2 hours of the clamp with sampling as described above. Steady‐state samples were taken at three time points in the final 30 minutes of each phase (basal, low insulin and high insulin). Rates of glucose production and glucose disposal were calculated using modified versions of the Steel equations.^20^, ^21^

#### 2.2.3 ^2^H_5_‐glycerol infusion

Whole body lipolysis was assessed, by measuring glycerol rate of appearance (Gly Ra) using stable isotope dilution methodology. ^2^H_5_‐glycerol (0.1 μmol/kg/min) (CK Gas Ltd) was infused throughout the duration of the clamp. The decrease in Gly Ra, from basal to low and high‐insulin, reflects the suppression of lipolysis by insulin. Regression analysis incorporating the circulating insulin concentrations at the various stages of the clamp was used to determine the insulin concentration needed to half‐maximally suppress Gly Ra (EC_50_).

#### 2.2.4 Subcutaneous adipose tissue (SAT) microdialysis and biopsy

A single microdialysis catheter (M Dialysis 63 40/30; Prospect Diagnostics ltd, Dronfield, UK) was inserted under local anaesthetic (1 mL of 1% lignocaine) into the subcutaneous adipose tissue 5 cm to lateral to the umbilicus. Microdialysis samples were taken at 30‐minute intervals for the duration of the two‐step clamp as we have described previously.^22^ Thirty minutes after starting the low‐dose insulin infusion, a subcutaneous abdominal adipose tissue biopsy was performed (contra‐lateral to the microdialysis catheter) using an aseptic technique under local anaesthetic (1‐2 mL of 1% lignocaine), in order to obtain approximately 250‐500 mg of adipose tissue. The sample was immediately snap frozen in liquid nitrogen and stored at −80°C for subsequent total RNA extraction, reverse transcription and real‐time PCR analysis.MRS, DXA protocol and isotope analysis are listed in Supporting Information S4 in Appendix S1.

### 2.3 Ethics

The study was conducted in accordance with the guidelines of the Declaration of Helsinki and the principles of Good Clinical Practice. All original study protocols had been reviewed and approved by the local National Research Ethics Service (NRES) Committee West Midlands, Solihull (East Midlands REC Centre) ethics committees REC reference 12/WM/0122&12/WM/028.

### 2.4 Statistics

Continuous clinical and laboratory variables are reported as mean and standard error (SE) since all variables showed parametric distribution on D'Agostino and Pearson Omnibus Normality testing. Categorical variables are reported as number and percentages. Area under the curve (AUC) analysis was performed using the trapezoidal method for interstitial glycerol release during the clamp. For comparison of single variables, paired Student's *t* tests were used (or nonparametric equivalents where data were not normally distributed). Where repeated samples were taken repeated‐measures one‐way ANOVA was used, incorporating Dunnett's test for multiple comparisons. The significance level was set at *P* < 0.05, and all analyses were performed using the GraphPad Prism 5.0 software package (GraphPad Company, San Diego, CA, USA).

## 3 RESULTS

### 3.1 Participants’ characteristics and systemic insulin sensitivity

Patient demographics, anthropometry and baseline biochemistry are presented in Supporting Information Table S5 in Appendix S1. In the CHC group, eight patients were infected with genotype (Gt)1 and five with Gt3 HCV. Serum cholesterol, triglycerides and creatinine were all lower in CHC patients compared to control subjects.

### 3.2 Differences in peripheral, hepatic and adipose tissue insulin sensitivity in CHC patients and control subjects

#### 3.2.1 Systemic insulin sensitivity and glucose disposal

**Figure 1:**
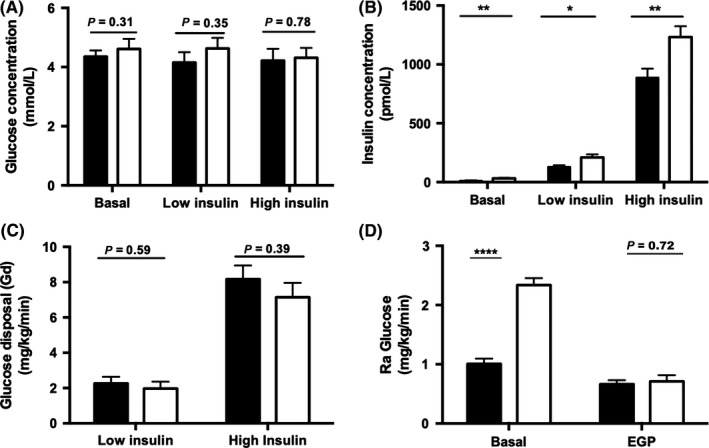
Hepatitis C virus infection increased systemic insulin resistance (IR), with limited effect on hepatic and peripheral IR. Circulating glucose (A) and insulin (B) levels were measured during the two‐step clamp. Muscle and hepatic IR was determined by suppression of glucose disposal, Gd (C) and rate of appearance, Ra (D). Black bar = healthy controls, White bar = CHC. ****P < 0.0001,**P < 0.01,*P < 0.05

Fasting glucose levels were similar in CHC and control subjects (4.39 ± 0.1 vs 4.7 ± 0.1 mmol/L, *P* = 0.08) and were successfully maintained across the clamp (Figure 1A). In contrast, fasting insulin and homoeostatic model assessment for insulin resistance (HOMA‐IR) were significantly increased in the CHC group consistent with systemic insulin resistance.

Despite insulin infusion rates being similar between the two groups, low‐ and high‐dose insulin concentrations were higher in the CHC group (Figure 1B). Glucose utilization under high‐dose insulin (M/I values) was lower in patients with CHC. However, body weight‐adjusted glucose disposal rates (Gd) were similar at low‐ and high‐dose insulin infusion (Figure 1C).

#### 3.2.2 Glucose production

Basal glucose production rates (Ra Glucose) were significantly higher in CHC patients (Figure 1D) although endogenous glucose production (EGP), reflecting hepatic insulin sensitivity and measured following low‐dose insulin infusion, was similar between CHC and healthy control subjects (0.71 ± 0.10 vs 0.66 ± 0.68 mg/kg/min, *P* = 0.72) (Figure 1D).

#### 3.2.3 Lipid metabolism

**Figure 2:**
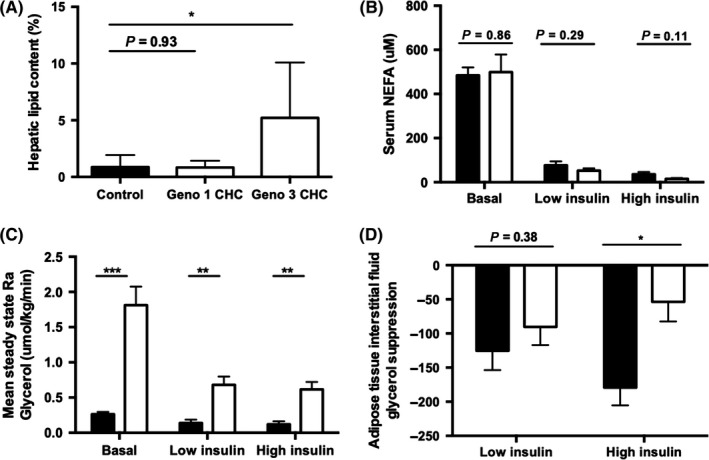
Hepatitis C virus infection increased adipose tissue insulin resistance Hepatic lipid content (by magnetic resonance spectroscopy/MRS) (A) and mean circulating nonesterified fatty acid (NEFA) concentrations (B), measured in controls and chronic hepatitis C. Whole body lipolysis measured by Ra(glycerol) (C). Change in mean levels of glycerol release from basal was determined to quantify the rate of lipolysis in subcutaneous adipose tissue at low‐ and high‐dose insulin infusion (D). *P < 0.05, **P < 0.01, ***P < 0.001

Proton density fat fraction measured by MRS that quantifies hepatic lipid content was similar between CHC patients and healthy control subjects (1.5 ± 0.7 vs 0.8 ± 0.3%, *P* = 0.33). However, if one excludes HCV Gt1 infected patients, the remaining Gt3 patients showed increased hepatic lipid content compared to the control subjects (5.2 ± 2.4 vs 0.8 ± 0.3%, *P* = 0.01) (Figure 2A).

Fasting circulating NEFA levels were similar between CHC patients and control subjects in the basal and insulinaemic states (Figure 2B). Ra glycerol, reflecting global lipolytic rates, was suppressed by insulin. It was higher in CHC compared to control subjects in the basal fasting state, after low‐dose and high‐dose insulin infusion consistent with an impaired ability of insulin to suppress lipolysis, largely reflecting adipose tissue insulin resistance (Figure 2C).

Adipose tissue microdialysis was used to examine the dynamic function of subcutaneous abdominal adipose tissue (SAT). Insulin suppressed adipose tissue interstitial fluid glycerol concentrations; however, the magnitude of this suppression following low‐dose insulin infusion was comparable between CHC patients and control subjects. However, endorsing our observations with systemic Ra Glycerol, high‐dose insulin failed to suppress abdominal SAT glycerol release in CHC (54 ± 29 vs 179 ± 26 μmol/kg/min, *P* = 0.013) (Figure 2D).

### 3.3 The impact of HCV eradication on liver chemistry, body composition and systemic insulin sensitivity

**Figure 3:**
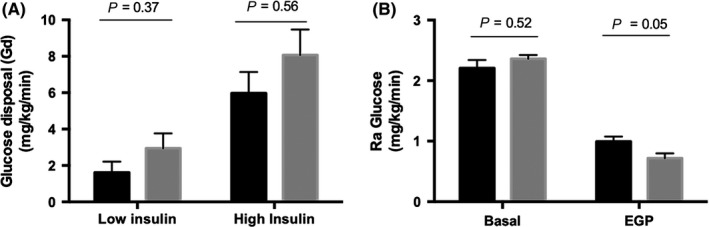
Viral eradication in chronic hepatitis C patients improves hepatic and adipose tissue insulin resistance. The degree of skeletal and hepatic insulin sensitivity was determined by suppression of Gd (A) and Ra glucose (B) following viral eradication

Body composition and metabolic parameters including BMI, weight, total and android/gynoid fat mass and serum lipids were unchanged before or after antiviral therapy. Alanine aminotransferase and aspartate aminotransferase improved after viral cure whilst gamma glutamyltransferase, bilirubin and albumin did not change (Supporting Information S6 in Appendix S1). Gd following both low‐ and high‐dose insulin infusion was unchanged after viral cure (Figure 3A).

### 3.4 Viral eradication in CHC patients improves hepatic and adipose tissue insulin resistance

Following viral eradication, EGP improved (0.99 ± 0.17 vs 0.72 ± 0.18 mg/kg/min; *P* = 0.05) consistent with increased hepatic insulin sensitivity (Figure 3B). Hepatic steatosis did not change following viral cure (4.217 ± 2.643 vs 2.893 ± 1.257, *P* = 0.67).

**Figure 4:**
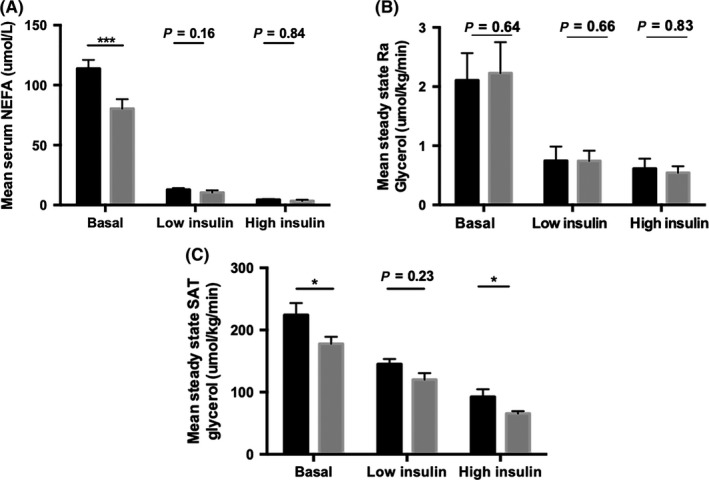
Sustained virological response improves global and subcutaneous adipose tissue (SAT) insulin sensitivity. Circulating nonesterified fatty acid (NEFA) concentrations (A) and Ra glycerol (B) were measured to determine global insulin resistance and whole body lipolysis. Mean levels of glycerol release during steady state were determined to quantify the rate of lipolysis in SAT.*P < 0.05, ***P < 0.001

Viral cure did not significantly alter the contribution of DNL to the circulating triglyceride pool (5.6 ± 4.3 vs 4.3 ± 4.7%, *P* = 0.68). Fasting circulating NEFA levels were reduced following viral cure (112 ± 42 vs 80 ± 39 μmol/L, *P* < 0.001). Although NEFA levels were suppressed by low‐ and high‐dose insulin infusions, there were no significant differences when comparing NEFA levels before and after viral eradication (Figure 4A).

Ra glycerol in the basal fasting state or after low‐ or high‐dose insulin fusion was not affected by viral cure (Figure 4B). Following viral cure, SAT interstitial glycerol release decreased in the fasting state, consistent with enhanced SAT insulin sensitivity (261 ± 128 vs 194 ± 43 μmol/kg/min, *P* < 0.05) (Figure 4C). However, there was no difference in abdominal SAT glycerol release after low‐dose insulin infusion (148 ± 57 vs 145 ± 62 μmol/kg/min, *P* = 0.23). Importantly, viral cure enhanced the ability of high‐dose insulin infusion to suppress local SAT glycerol release (99 ± 23 vs 71 ± 25 μmol/kg/min, *P* < 0.05), independent of CHC genotype (Figure 4C).

### 3.5 HCV cure increases transcript levels of lipogenic and insulin signalling genes in subcutaneous adipose tissue

**Figure 5:**
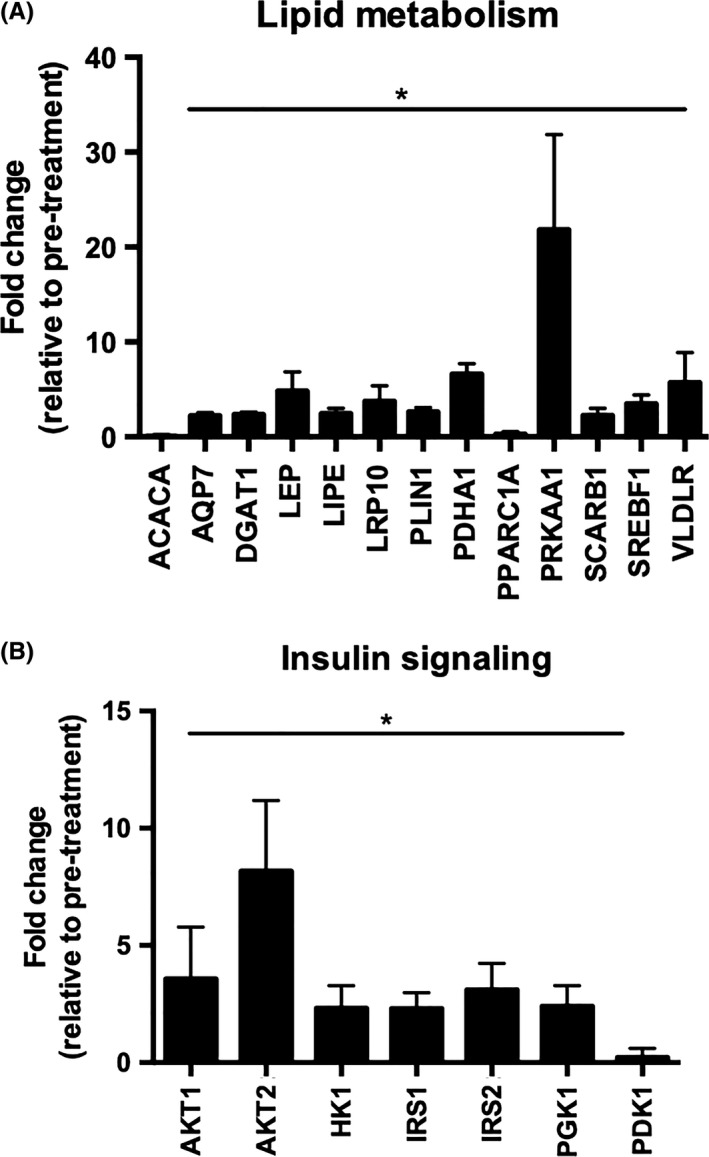
Hepatitis C virus eradication regulates the mRNA expression of metabolic genes. Fold change of 13 lipogenic (A) and 7 insulin signalling genes (B) with significant differences in expression levels in pre‐ and postviral eradication. Data are presented as mean ± SE fold induction compared to untreated cells (quantified relative to GAPDH), *P < 0.05

mRNA levels of genes involved in the metabolic pathways including insulin sensitivity and lipid metabolism were analysed in SAT using a Fluidigm BioMark HD System 96.96 Dynamic Array, San Francisco, CA, USA 34 of the 58 genes showed significant differences in expression levels before and after viral eradication; 13 were involved in lipid metabolism (Figure 5A) and 7 in insulin signalling (Figure 5B) (Supporting Information S3 in Appendix S1). Specifically, mRNA levels of diacylglycerol O‐acyltransferase‐1 (DGAT1), leptin (LEP) and sterol regulatory element binding transcription factor‐1 (SREBF1), the major regulator of lipogenic gene expression, were increased (2.4‐,4.8‐ and 3.5‐fold, respectively). mRNA expression levels of insulin receptor substrate (IRS)‐1 and ‐2 and p‐akt murine thymoma viral oncogene homologue (AKT)‐1 and ‐2 increased 3.6‐, 8.1‐, 2.3‐ and 3.1‐fold, respectively, consistent with our observations of enhanced insulin action after antiviral therapy. mRNA expression of hydroxysteroid 11‐β (HSD11β1) and dual specificity phosphatase 1 (DUSP1) were also increased (5.9‐ and 2.3‐fold, respectively). Taken together, these data suggest a shift towards safe lipid storage with less mobilization in the setting of enhanced insulin action postviral cure.

## 4 DISCUSSION

We have demonstrated that CHC is associated with an adverse metabolic phenotype characterized by systemic, hepatic and adipose tissue IR This is the first longitudinal study that has measured both carbohydrate and lipid flux in HCV‐infected patients before and after viral cure and demonstrates that the improvement in IR upon successful viral eradication was specific to hepatic and subcutaneous adipose tissue with limited alterations in body composition.

The association between HCV infection and T2DM is independent of the presence of cirrhosis.^23^ In vitro studies have demonstrated that HCV hijacks the lipid‐producing machinery of hepatocytes for its benefit, resulting in hepatic steatosis and insulin resistance. However, liver cirrhosis (regardless of aetiology) is also associated with insulin resistance.^24^, ^25^ The mechanisms by which these occur are out with the scope of this study. In order to examine tissue‐specific insulin sensitivity in CHC, only patients without cirrhosis were included to avoid the confounding effect of cirrhosis on insulin resistance.

Our CHC cohort had lower serum triglyceride levels compared to control, despite being well nourished and of normal BMI. This finding is similar to the observations by Dai et al.^26^ The association between HCV and hypobetalipoproteinaemia,^27^, ^28^ in part, may explain the observation. However, we did not observe increase in triglyceride level following viral eradication. There was significant hyperinsulinaemia in the CHC group compared to control subjects in this study. In patients with CHC, the presence of steatosis or advanced fibrosis was reported to associate with a reduction in hepatic insulin clearance.^29^ Reduction in insulin clearance plays an important role in the compensatory response to insulin resistance, and this was associated with increased hepatic lipase activity.^30^ As our study excluded patients with advanced fibrosis and significant steatosis, it is possible that HCV may mediate hepatic insulin clearance directly, and further studies are needed to delineate the underlying mechanisms.

Our detailed metabolic phenotyping approach has allowed us to dissect, in a tissue‐specific manner, the contribution of individual tissues to the systemic insulin resistance reported in HCV‐infected patients. Even though liver is the major reservoir supporting viral replication, the sites of insulin resistance in CHC have been debated in the published literature.^6^, ^8^ Some studies have suggested that the liver is the major site of insulin resistance^6^, ^7^ whilst others have implicated adipose tissue and skeletal muscle.^8^ The mechanisms underlying the crosstalk between liver and extra‐hepatic sites also remain largely unknown. The majority of studies published to‐date have used HOMA‐IR to quantify insulin resistance in CHC^31^, ^32^ which only provides a global, systemic measure of insulin sensitivity and does not discriminate between hepatic and peripheral effects.^33^

Despite inducing more steatosis and fibrosis, it is unclear whether the degree of insulin resistance is linked to the infecting HCV genotypes. The published evidence is controversial, with some suggesting higher insulin resistance among patients with Gt1^34^ whilst others show a higher prevalence in Gt3 infection.^35^ Some authors have reported data showing a similar prevalence of insulin resistance in both genotypes.^8^, ^36^ Even though our study was not powered to compare the two groups, we observed no difference in baseline hepatic or skeletal insulin resistance between patients infected with Gt1 or 3 CHC, or between healthy controls and CHC.

To the best of our knowledge, this is the first study to demonstrate the significance of SAT adipose tissue dysfunction in CHC. This has been linked to other liver conditions, especially nonalcoholic fatty liver disease.^22^ In our study, patients with CHC had adipose tissue insulin resistance as measured by serum NEFA concentrations, Ra glycerol and adipose tissue interstitial fluid glycerol release. One study explored the effect of HCV infection on whole body lipolysis.^6^ Vanni et al measured serum NEFA levels and Ra glycerol following ^2^H_5_‐glycerol infusion and found no difference in whole body lipolysis between healthy volunteers and patients with CHC. Some have suggested an association between HCV infection and alterations in adipocytokines, but data have been inconclusive.^37^, ^38^ Adipose insulin resistance as measured by failed suppression of circulating NEFA levels resulting in an increase in NEFA delivery and availability in the liver and can fuel lipid accumulation. However, both circulating NEFA levels and Ra glycerol measurements detect whole body lipolysis and are unable to correlate whole body lipolysis to a specific tissue. By pairing these observations with abdominal SAT microdialysis to measure mean steady‐state adipose interstitial fluid glycerol concentrations, we showed that patients with CHC had increased abdominal SAT‐specific insulin resistance compared to BMI‐ and age‐matched controls.

Upon successful viral cure, systemic insulin resistance did not alter, but hepatic and peripheral IR improved in patients with CHC. This was not accompanied by alterations in body weight or in hepatic lipid content. The improvement in hepatic IR was more pronounced in Gt3 infected patients (data not shown), which was perhaps unsurprising, as Gt3 HCV is known to be more pro‐steatogenic (indeed, hepatic lipid content was higher in the Gt3 patients in our study), and therefore, its eradication would be expected to improve hepatic steatosis and hence hepatic IR. More interestingly, the improvement in skeletal IR was only observed in Gt1 CHC. However, we acknowledged that this study was not powered to study the genotype‐specific contribution to insulin resistance.

Sustained virological response significantly improved SAT insulin sensitivity in both Gt1&3 CHC as evidenced by interstitial fluid glycerol suppression by insulin. The observed beneficial impact on adipose function could mediate a reduction in FFA delivery to the liver resulting in less hepatic steatosis. Even though the mechanisms that drive the improvement in adipose tissue function are unknown, we have shown that they are occur independent of the changes in body composition of fat mass. Taken together, our data show that there are tissue‐specific effects of viral eradication, namely in liver and fat, but not in skeletal muscle and our gene expression data from adipose tissue would appear to endorse this.

Our study is not without limitations. Comparison between a mixed gender CHC cohort and a purely male control cohort may have underestimated the degree of insulin resistance in the CHC group, as male subjects are perceived to have higher insulin resistance due to the greater amounts of visceral and hepatic adipose tissue, and lack the protective effect of oestrogen.^39^ The number of participants is small making it difficult to extrapolate the relevance of observed metabolic perturbation in patients with CHC or the subsequent improvements in tissue‐specific insulin sensitivity after treatment. The small number of participants also limits the interpretation of our data and mean that the analysis of HCV treatment and Gt in inducing IR and hepatic steatosis is likely to be underpowered. Visceral adipose tissue function is perhaps the most critical determinant of NEFA delivery to the liver, but real‐time assessments of visceral adipose tissue function are not feasible in human studies and therefore we have had to extrapolate our findings from the SAT depot. Not all CHC patients received the same antiviral therapies. Pegylated interferon has been shown to alter insulin sensitivity both in vivo and in vitro.^40^, ^41^ Whilst we included a washout period of 3‐6 months in our study design to eliminate any direct effect(s) of interferon treatment, we did observe a more dramatic improvement in systemic insulin sensitivity in patients who did not receive interferon, but without any change in body composition or BMI.

## 5 CONCLUSIONS

This study demonstrated that CHC is intricately linked to insulin resistance and hepatic steatosis. Using state‐of‐the‐art metabolic assessments, we have demonstrated that both hepatic and adipose tissue insulin sensitivity improved after viral eradication. The identification of extra‐hepatic effects of HCV infection, especially in the adipose tissue is novel and has important clinical relevance. Further studies are needed to evaluate the potential interaction between HCV and adipose tissue in inducing insulin resistance and the genotype‐specific mechanisms involved, to allow for novel and targeted therapies.

## CONFLICT OF INTEREST

Nothing to declare.

## AUTHOR'S CONTRIBUTIONS

TRL drafted the article and contributed to the conception and design of the study. JMH, AIO, MJA, SFA, NPD, RF contributed to the design of the study, generation and analysis of the data and final approval of the submitted version. DJM, JAM and JWT contributed to the interpretation of the data and final approval of the version.
