# Supplementary material for: Hepatitis C virus infection is associated with hepatic and adipose tissue insulin resistance that improves after viral cure
Source: Clin Endocrinol (Oxf). 2019 Jan 17;90(3):440–8. doi: 10.1111/cen.13924 (PMC6446809; doi:10.1111/cen.13924)
Supplement: Supplementary file 1 [file CEN-90-440-s001.docx]

**Supplementary data**

***S1.*** *Inclusion and exclusion criteria of study subjects*

| **Inclusion criteria** | **Exclusion criteria** |
| --- | --- |
| Chronic hepatitis C cohort:  Treatment-naive patients anti-HCV positivity and detectable levels of HCV RNA, aged 18-65 years, body mass index (BMI) between 18-34kg/m^2^ and due to receive anti-viral therapy within 6 months.  Healthy control subjects:-  Male volunteers aged 18–65 years, non-diabetic and normotensive, and had a BMI between 20 and 34kg/m^2^. All consumed alcohol within recommended limits and had normal liver function tests. | Participants were excluded if they had co-existing liver diseases of other aetiology, cirrhosis (from imaging or histology), diabetes mellitus, recent or concomitant drug use of inducers of hepatic steatosis/weight-inducing therapy or a history of excess alcohol consumption (>14 units per week). |

***S2****.* ***Types and duration of antiviral treatment.***


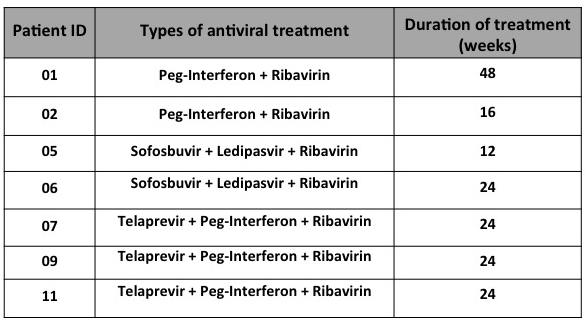


*Peg=pegylated, SVR=sustained virological response. Telaprevir=protease inhibitor, Sofosbuvir=NS5B polymerase inhibitor, Ledipasvir=NS5A inhibitor.*

***S3. Study design***

**
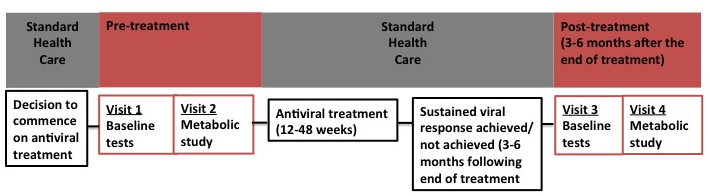
**

***S4. Clinical protocol and analysis***

***MRS and DXA protocol:*** MRS of the liver was performed on a Siemens Verio 3T MRI system using single-voxel PRESS-MRS, following orthogonal T1w VIBE (3D-GRE) breath-hold localisers of the thorax and abdomen. Image-based shimming was performed over a co-localised (40x40x40) mm^3^ region, with manual adjustments where necessary to achieve water line width < 40Hz. Spectra were fitted offline using Tarquin^4^ with a customised basis set containing lipid and metabolite peaks. Strict quality control (QC) criteria were applied and visual inspection of fits was performed. Exponential fitting was performed to estimate water, lipid and metabolite T2s and lipid and metabolite levels were calculated relative to water at each echo time (TE) with and without T2 correction. DXA scan uses two X-ray energies to measure the presence of bone mineral, lean tissue and adipose tissue. DXA was employed for body composition assessment using Hologic Discovery/W DXA (software version Apex 3.0, Hologic Inc). Specific fat phenotype was measured using android, gynecoid, peripheral (arms and legs), and trunk regions of interest, with subsequent calculation of android:gynecoid and trunk:peripheral fat ratios (Gregson et al., 2013).

***Biochemical and stable isotope analysis:***

*Clinical and Biochemical Parameters.* Participant demographics and clinical/biochemical measures were recorded at the study visit. Blood counts, urea, creatinine, electrolytes, cholesterol, triglycerides, liver chemistry, glucose and HbA1C were measured using standard laboratory methods (Roche Modular system, Roche Ltd, Lewes, UK). Insulin was measured using commercially available colorimetric ELISA (Mercodia, Uppsala, Sweden) with an in-house coefficient of variation of <5%. Serum NEFAs were measured using commercially available kits according to the manufacturer’s instructions (ZenBio, NC, US).

*Stable Isotope Mass Spectrometry Analysis.* The enrichment of U-[^13^C]-glucose in plasma was determined by gas chromatography–mass spectrometry (model 5973; Agilent technologies, Cheshire, UK). Deuterium enrichment of the body water pool was measured using the Gasbench II (www.thermo.com), an automated H2/H2O equilibration device, coupled on-line to a ThermoFinnigan Deltaplus XP Isotope Ratio Mass Spectrometer (IRMS; ThermoFinnigan MAT GmbH, Bremen, Germany). Detailed methods have been previously described [28]. Deuterium enrichment in the palmitate fraction of total plasma triglycerides was measured on an automated GC/TC/IRMS system (ThermoFinnigan Delta Pus XP; [www.thermo.com](http://www.thermo.com)).

*Abdominal SAT Microdialysis.* Microdialysate samples were analysed using a mobile photometric, enzyme-kinetic analyser (CMA Iscus Flex) for glycerol concentration as a reflection of local adipose tissue lipolysis.

*Contribution of Hepatic DNL to Total Palmitate Synthesis.* The percentage contribution of hepatic DNL to endogenous palmitate synthesis was determined by the incorporation of ^2^H_2_O in the palmitate present in the plasma total triglyceride pool, as previously described (40). This percentage was calculated from the increase in the ^2^H/^1^H ratio in the palmitate methylester of the total triglyceride fraction and in the water of plasma samples taken before (17:00 hours, at admission) and 14 h after the initial ingestion of the ^2^H_2_O tracer (08:00 hours, before the start of the hyperinsulinaemic euglycaemic clamp). The following formula was used: %hepatic DNL contributes to endogenous palmitate synthesis=(delta ^2^H/^1^H ratio in palmitate methylester)/(delta ^2^H/^1^H ratio in waterpool) x (34/22) x 100%. In the equation, 34 is the total number of H-atoms in palmitate methylester and 22 is the number of water molecules incorporated into palmitate via DNL as observed in previous rodent studies (41) and currently used in human studies (42).

***Gene expression analysis:***

*RNA isolation and cDNA synthesis.* Total RNA from blood samples was extracted using Roche High Pure PCR Template Preparation Kit (Catalog number; 11796828001) according to the manufacturer's instructions. Total RNA was reverse-transcribed into first-strand complementary DNA (cDNA) using a High Capacity cDNA Reverse Transcription Kit (Invitrogen, Life Sciences, Catalog Number: 4368814). The cycle conditions were as follows: 10 min at 25 °C, 120 min at 37 °C, and 5 min at 85 °C.

*High-throughput quantitative PCR.* qRT-PCR was performed in two steps after total RNA purification and conversion to single stranded cDNA using polyT priming: The targeted genes were pre-amplified in a single 14-cycle PCR reaction for each sample by combining 100 ng cDNA with the pooled primers and TaqMan Pre-Amp Mastermix (Fluidigm BioMark™) following conditions recommended in the manufacturer's protocol, and 21 Å~ 84 (samples Å~ primers) qRT-PCR reactions were performed for each primer pair on each sample on a 96.96 array using the EvaGreen detection assay following standard Fluidigm protocols. Ct values were calculated from the system software [BioMark Real-Time PCR Analysis, Fluidigm]. Primer sets amplifying the mRNAs of the relevant genes are presented below.

| **Target** | **Gene Full Name** | **FP** | **RP** | **Fold change (mean±SD)** | **p value** |
| --- | --- | --- | --- | --- | --- |
| ABHD5 | abhydrolase domain containing 5 | GCAGCATTGACTCCCTTTAACC | AGGCCTTAAACGCTGCACTA | 1.40±0.32 | 0.12 |
| ACACA | acetyl-CoA carboxylase alpha | ATCCCAGCTGATCCAGCAAA | GCAGAATCTGGGAACCAAACC | 0.08±0.07 | **<0.0001** |
| ADIPOQ | adiponectin, C1Q and collagen domain containing | CCTGGTGAGAAGGGTGAGAAA | GGTTTCACCGATGTCTCCCTTA | 1.45±0.36 | 0.22 |
| ADRB2 | adrenoceptor beta 2 | ATGGACTCCGCAGATCTTCC | AAGTGCCCATGATGATGCCTA | 2.36±0.31 | **<0.0001** |
| AKT1 | v-akt murine thymoma viral oncogene homolog 1 | CACACACTCACCGAGAACC | TCGTGGGTCTGGAAAGAGTA | 3.56±1.11 | **0.01** |
| AKT2 | v-akt murine thymoma viral oncogene homolog 2 | ACGGGGCCACCATGAAAA | GGCCATAGTCATTGTCCTCCA | 8.17±3.01 | **<0.001** |
| APOE | apolipoprotein E | CCCAGGTCACCCAGGAAC | TGTTCCTCCAGTTCCGATTTGTA | 1.73±1.03 | 0.41 |
| AQP7 | aquaporin 7 | GACAAAACATGGTTCAAGCATCC | CTATCACGGACCAGGAGACC | 2.23±0.34 | **0.002** |
| ARNT | aryl hydrocarbon receptor nuclear translocator | CTGTGTGGCTACTGTTGGCTA | GCTGGTCTTCAGGATGACAGAA | 1.29±0.11 | **0.04** |
| CCL2 | chemokine (C-C motif) ligand 2 | TAGCAGCCACCTTCATTCCC | CCTCTGCACTGAGATCTTCCTA | 3.14±1.41 | **0.009** |
| CD36 | CD36 molecule (thrombospondin receptor) | AGCAGCAACATTCAAGTTAAGCA | GCGTCCTGGGTTACATTTTCC | 2.49±0.71 | 0.06 |
| CD81 | CD81 molecule | GGCAGCAACATCATCAGCAA | AGCAATGCCGATGAGGTACA | 2.25±0.47 | **0.02** |
| CIDEC | cell death inducing DFFA like effector c | TTGGCTGCCTGAACGTGAA | TGTGGCCTGCATGCTGAA | 4.60±2.27 | **0.03** |
| CPT1A | carnitine palmitoyltransferase 1A (liver) | TCCATGCCATCCTGCTTTACA | AGTGGAATCGTGGATCCCAAA | 1.48±0.47 | 0.34 |
| DGAT1 | diacylglycerol O-acyltransferase 1 | ACTACCGTGGCATCCTGAAC | GAAATAACCGGGCATTGCTCAA | 2.36±0.16 | **<0.0001** |
| DUSP1 | dual specificity phosphatase 1 | AGACATCAGCTCCTGGTTCA | CAGTGGACAAACACCCTTCC | 5.86±2.60 | **0.003** |
| FABP4 | fatty acid binding protein 4 | ATGTGTGATGCTTTTGTAGGTAC | CCACTTTCCTGGTGGCAAA | 2.94±1.52 | 0.22 |
| FABP5 | fatty acid binding protein 5 | GACGCAGACCCCTCTCTG | TTCCTTCCAGCTGCTGAACT | 5.26±2.36 | 0.05 |
| FASN | fatty acid synthase | GGAGGGGACAGTGCATCAA | GTTTACACTCCTCCCAGGACAA | 6.87±4.35 | 0.17 |
| GAPDH | glyceraldehyde-3-phosphate dehydrogenase | GAACGGGAAGCTTGTCATCAA | ATCGCCCCACTTGATTTTGG | 0.99±0.00 | - |
| GLUL | glutamate-ammonia ligase | GTCAAGATTGCGGGGACTAA | CCCATGCTGATTCCTTCACA | 2.38±0.78 | 0.10 |
| HIF1A | hypoxia inducible factor 1, alpha subunit | CAGTCGACACAGCCTGGATA | TTCTTCTGGCTCATATCCCATCAA | 2.43±0.48 | **0.005** |
| HIF3A | hypoxia inducible factor 3, alpha subunit | CTCCTTGCGCATGAAGAGTA | CTCATATGTCCAGAGCAGTTCA | 0.60±0.30 | 0.06 |
| HK1 | hexokinase 1 | TCATTTCCCTGCCAGCAGAC | CGCAGTCTGTTGCCTTAAAACC | 2.32±0.68 | **0.003** |
| HSD11B1 | hydroxysteroid (11-beta) dehydrogenase 1 | GAAGCAGAGCAATGGAAGCA | TTGCAGAATAGGCAGCAACC | 2.30±0.80 | **0.03** |
| IFNA1 | interferon, alpha 1 | TGACTCATACACCAGGTCAC | CAGGGGTGAGAGTCTTTGAA | 3.01±2.22 | 0.17 |
| IRS1 | insulin receptor substrate 1 | CAGAAGCAGCCAGAGGAC | AGAGGATTTGCTGAGGTCATTTA | 2.30±0.68 | **0.01** |
| IRS2 | insulin receptor substrate 2 | TGTCCCACCACTTGAAGGAG | TGACATGTGACATCCTGGTGAT | 3.11±1.12 | **0.01** |
| JAK2 | Janus kinase 2 | TCTGCAGTGGAGGAGATAAACC | TGCAGGAAGCTGATGCCTA | 7.65±3.75 | **0.02** |
| LEP | leptin | CACCAAAACCCTCATCAAGACAA | AGCCCAGGAATGAAGTCCAA | 4.81±2.05 | **0.03** |
| LIPE | lipase, hormone-sensitive | AGTTAAGTGGGCGCAAGTCC | GCCAGTGCTGCTTCAGACA | 2.43±0.60 | **0.02** |
| LOX | lysyl oxidase | ATCCAGGCGTCCACGTAC | AGCAGCACCCTGTGATCATAA | 1.99±1.45 | 0.26 |
| LPL | lipoprotein lipase | TGGCCGAGAGTGAGAACA | AGCTTCAACATGAGTAGTTCTCC | 4.92±2.20 | 0.09 |
| LRP10 | LDL receptor related protein 10 | GCAGCAAGGAACAGACTGTCA | GAGAGGGGAGCGTAGGGTTA | 3.76±1.64 | **0.006** |
| NR3C1 | nuclear receptor subfamily 3 group C member 1 | GCAGCAGTGAAATGGGCAAA | CAGTAGGGTCATTTGGTCATCCA | 2.03±0.70 | 0.11 |
| OCLN | occludin | AACTGGCGGCGAGTCC | TCCTGTAGGCCAGTGTCAAAA | 2.26±1.60 | 0.22 |
| PDHA1 | pyruvate dehydrogenase (lipoamide) alpha 1 | GTGCTGGTAGCATCCCGTAA | CCTTCTTCCAGCCGGTGAA | 6.61±1.10 | **<0.0001** |
| PDK1 | pyruvate dehydrogenase kinase 1 | ACCAAGACCTCGTGTTGAGAC | AAGACGTGATATGGGCAATCCA | 0.22±0.22 | **<0.001** |
| PDK4 | pyruvate dehydrogenase kinase, isozyme 4 | CTACTCGGATGCTGATGAACCA | CCAATGTGGCTTGGGTTTCC | 0.22±0.22 | **<0.001** |
| PER1 | period circadian clock 1 | TGATTGCAGAGCGCATCCA | TGTGTGCCGCGTAGTGAAA | 4.10±1.09 | **0.001** |
| PGK1 | phosphoglycerate kinase 1 | GTGGAATGGCTTTTACCTTCC | CTTGGCTCCCTCTTCATCAA | 2.41±0.88 | 0.11 |
| PIK3CA | phosphatidylinositol-4,5-bisphosphate 3-kinase catalytic subunit alpha | CTGCAGTTCAACAGCCACAC | ACAGGTCAATGGCTGCATCA | 1.44±0.53 | 0.19 |
| PLIN1 | perilipin 1 | TCACCTTGCTGGATGGAGAC | ATTCGCAGGTGCCACTCA | 2.61±0.47 | **0.005** |
| PLIN2 | perilipin 2 | CCTCTCATGGGTAGAGTGGAA | GCAATTGCAAGAGTACGTGAC | 5.94±4.01 | 0.21 |
| PPARGC1A | PPARG coactivator 1 alpha | ACTTTTGTGGACGCAAGCAA | TGGAAGCAGGGTCAAAGTCA | 0.28±0.07 | **<0.001** |
| PPARG | peroxisome proliferator-activated receptor gamma | TAGATGACAGCGACTTGGCAATA | TGGGCTTCACATTCAGCAAAC | 1.92±0.47 | 0.06 |
| PPIA | peptidylprolyl isomerase A (cyclophilin A) | TCTGGTTCCTTCTGCGTGAA | CCAGGGAATACGTAACCAGACA | 3.46±1.36 | 0.05 |
| PRKAA1 | protein kinase, AMP-activated, alpha 1 catalytic subunit | CCAACTATGCTGCACCAGAA | AGAATAACCCCACTGCTCCA | 21.82±10.0 | **0.008** |
| RAB18 | RAB18, member RAS oncogene family | GCTAACCACCCTGAAGATCC | TGCAAGTTCTGGATCAAACGTA | 2.30±0.12 | **<0.0001** |
| SCARB1 | scavenger receptor class B member 1 | GAGATCCTGAAGGGCGAGAA | GATGTTGCTTTTGTGCCTGAAC | 2.27±0.75 | **0.04** |
| SIRT1 | sirtuin 1 | ACAAAGTTGACTGTGAAGCTGTAC | GTTCATCAGCTGGGCACCTA | 1.79±0.11 | **<0.0001** |
| SLC2A4 | solute carrier family 2 (facilitated glucose transporter), member 4 | TTCTCCAACTGGACGAGCAA | GGACCGCAAATAGAAGGAAGAC | 1.25±0.69 | 0.55 |
| SOCS1 | suppressor of cytokine signaling 1 | CATCCGCGTGCACTTTCA | GCTCGAAGAGGCAGTCGAA | 1.42±1.26 | 0.45 |
| SRD5A1 | steroid-5-alpha-reductase, alpha polypeptide 1 | GCCATGTTCCTCGTCCACTA | CAACAGTGGCATAGGCTTTCC | 1.89±1.43 | 0.32 |
| SREBF1 | sterol regulatory element binding transcription factor 1 | CAGCAACCAGAAACTCAAGCA | GCCGACACCAGATCCTTCA | 3.48±0.95 | **0.006** |
| UCP2 | uncoupling protein 2 (mitochondrial, proton carrier) | TTCCTCTGGATACTGCTAAAGTCC | TCAGAATGGTGCCCATCACA | 3.26±1.37 | **0.02** |
| VEGFA | vascular endothelial growth factor A | GAGGAGGGCAGAATCATCAC | GTCTCGATTGGATGGCAGTA | 2.90±0.98 | **0.02** |
| VLDLR | very low density lipoprotein receptor | CCTAGCTCATCCTCTTGCACTA | TGGCACCATAGACTGCTTCA | 5.71±1.82 | **0.003** |

List of 58 genes involved in metabolism and inflammation and the primer sets amplifying the mRNAs of the relevant genes. Fold change (relative to pre-treatment) expressed as mean±SD.

***S5. Participant characteristics (healthy volunteers vs. CHC).***

Data are n (%) or mean (SD). CHC=chronic hepatitis C, BMI=body mass index, HDL=high density lipoprotein, Ra glucose= glucose production rates, EGP=endogenous glucose production, Gd=glucose disposal. Student’s t-test.

***S6. Participant’s characteristics of patients with CHC before and after SVR***

Data are n (%) or mean (SD). IFN=interferon, BMI=body mass index, HOMA-IR=homeostasis model assessment-estimated insulin resistance. HDL=high density lipoprotein, PDFF=proton density fat fraction. ALT=alanine aminotransferase, AST=aspartate aminotransferase, GGT=gamma glutamyltransferase, Ra glucose= glucose production rates, EGP=endogenous glucose production, Gd=glucose disposal. Student’s t-test.
